# Associations of C-reactive Protein with 25-hydroxyvitamin D in 24 Specific Diseases: A Cross-sectional Study from NHANES

**DOI:** 10.1038/s41598-020-62754-w

**Published:** 2020-04-03

**Authors:** Fang Yang, Mengzi Sun, Chong Sun, Jiagen Li, Xiuning Yang, Chunli Bi, Min Wang, Liyuan Pu, Jianmeng Wang, Chunxiao Wang, Meizhen Xie, Yan Yao, Lina Jin

**Affiliations:** 1 Department of Health Management Center, the First Hospital of Jilin University, Changchun, Jilin, 130021 China; 2 Key Laboratory of Organ Regeneration and Transplantation of Ministry of Education, School of Public Health, Jilin University, Changchun, Jilin, 130021 China; 3 Department of Hepatobiliary Surgery, Affiliated hospital of Beihua University, Jilin, Jilin, 132011 China; 4 Department of Geriatrics, the First Hospital of Jilin University, Changchun, Jilin, 130021 China; 5 Department of Clinical Medicine, School of Clinical Medicine, Changchun, Jilin, 130021 China

**Keywords:** Preventive medicine, Epidemiology

## Abstract

Most diseases might be associated with acute or chronic inflammation, and the role of vitamin D in diseases has been extensively explored in recent years. Thus, we examined the associations of one of the best markers for inflammation ― C-reactive protein (CRP) with 25-hydroxyvitamin D [25(OH)D] in 24 specific diseases. We performed cross-sectional analyses among 9,809 subjects aged ≥18 years who participated in the U.S. National Health and Nutrition Examination Survey (NHANES) in 2007~2010. The generalized additive model (GAM) was used to explore the associations of CRP with 25(OH)D in different diseases, adjusted for the age, gender, examination period and race. Distributions of CRP were significantly different (*P* < 0.05) in gender, examination period and race, and distributions of 25(OH)D were different (*P* < 0.05) in the examination period and race. Generally, CRP was negatively associated with 25(OH)D for majority diseases. 25(OH)D was negatively associated with CRP generally, and the associations were disease-specific and disease category-specific. In respiratory, gastrointestinal and mental diseases, the associations tended to be approximately linear. While in metabolic diseases, the associations were nonlinear, and the slope of the nonlinear curve decreased with 25(OH)D, especially when 25(OH)D < 30 μg/L.

## Introduction

The exploration and understanding of disease mechanisms involved lots of aspects, for instance, the steady state decomposition process of metal ions was related to numerous diseases^1^, free radicals and related active substances could affect health as a mediator of tissue damage and disease^2^, defects in pre-mRNA splicing were proven to be a common pathogenic mechanism^3^, etc. In recent years, with the expanding understanding of diseases, the role of vitamin D has also been extensively explored. Vitamin D not only affected the bone metabolism regulation and female reproductive and pregnancy outcomes^4^, but also had potential correlation to cancer^5,6^. Moreover, vitamin D was also considered to have potential beneficial effects on reducing inflammation and alleviating pain^7,8^. Therefore, exploring the role of vitamin D in common diseases is interesting and meaningful.

Previous studies suggested that most diseases might be associated with acute or chronic inflammation to some extent^9–11^. Insufficient inflammation could lead to persistent infection of pathogens, while excessive inflammation may contribute to chronic or systemic inflammatory diseases^12^. C-reactive protein (CRP) was considered as one of the best markers for measuring inflammation caused by bacterial infection or tissue damage, which was produced by stimulation of interleukin-1 (IL-1) and interleukin-6 (IL-6) in liver^13,14^. Therefore, it is meaningful to explore the role of vitamin D in common diseases via the associations of CRP with vitamin D.

A study of 923 patients in Netherlands found that vitamin D was negatively associated with CRP in both inflammatory and non-inflammatory diseases, and the liner associations were stronger in inflammatory diseases^15^. However, a randomized placebo-controlled trial of 413 patients in Australia showed that the associations of CRP with vitamin D were not significant^16^. There is still no consensus on the association between vitamin D and CRP, and further exploration is needed. Few studies focused on the nonlinear associations between vitamin D and CRP, and vitamin D was usually considered as a categorical variable classified as deficiency or not^8,17^. However, categorical variable cannot reflect the overall distribution of vitamin D, and cannot fully explore the associations of CRP with vitamin D.

It is a challenge to explore the associations of CRP with vitamin D by the overall distribution of vitamin D rather than by the average level of vitamin D through conventional methods (e.g., logistic regression). Fortunately, the generalized additive model (GAM) had great potential in addressing associations of continues variables. We therefore applied GAM to explore the associations of CRP with 25(OH)D in 24 specific diseases based on 9,809 participants who at least had one of diseases in the 2007~2010 in the U.S. National Health and Nutrition Examination Survey (NHANES).

## Results

### Descriptive characteristics of subjects with the 24 specific diseases

A total of 9,809 participants (4,703 males, 5,106 females) were involved in this study, and the characteristics of subjects with the 24 specific diseases were showed in Table 1, respectively.

**Table 1:** Characteristics of subjects with the 24 specific diseases (x ± S/n (%)).

Diseases	N	Age	Gender				
			Male	Female			
Asthma	847	49 ± 19	309 (36.5)	538 (63.5)			
Chronic bronchitis	294	58 ± 16	100 (34.0)	194 (66.0)			
Cold/ Chest cold	1687	47 ± 18	798 (47.3)	889 (52.7)			
Flu/ Pneumonia/ Ear infection	422	47 ± 17	182 (43.1)	240 (56.9)			
Emphysema	258	65 ± 13	153 (59.3)	105 (40.7)			
Stomach/ Intestinal illness	811	47 ± 18	334 (41.2)	477 (58.8)			
Diabetes	1268	62 ± 13	638 (50.3)	630 (49.7)			
High cholesterol	3274	59 ± 14	1606 (49.1)	1668 (50.9)			
Thyroid problem	746	61 ± 16	146 (19.6)	600 (80.4)			
Gout	482	65 ± 13	355 (73.7)	127 (26.3)			
Obesity	3919	48 ± 18	1918 (48.9)	2001 (51.1)			
Kidney stone	944	57 ± 16	552 (58.5)	392 (41.5)			
Failing kidney	277	59 ± 17	138 (49.8)	139 (50.2)			
Anemia	471	55 ± 19	104 (22.1)	367 (77.9)			
Liver condition	148	55 ± 14	71 (48.0)	77 (52.0)			
Heart attack	467	66 ± 13	322 (68.9)	145 (31.1)			
CHD	435	68 ± 11	307 (70.6)	128 (29.4)			
Stroke	393	67 ± 13	194 (49.4)	199 (50.6)			
Anxious	5601	46 ± 18	2446 (43.7)	3155 (56.3)			
Sleeping trouble	2566	53 ± 17	1044 (40.7)	1522 (59.3)			
Depression	126	51 ± 19	55 (43.7)	71 (56.3)			
Cancer	1022	65 ± 15	485 (47.5)	537 (52.5)			
Arthritis	2920	62 ± 14	1225 (41.9)	1695 (58.1)			
Prostate disease	51	68 ± 10	51 (100.0)	—			
**Diseases**	**Race**					**Examination period**	
	**Mexican American**	**Other Hispanic**	**Non-Hispanic White**	**Non-Hispanic Black**	**Other Races**	**Nov.1 ~ Apr.30**	**May.1 ~ Oct.31**
Asthma	74 (8.7)	98 (11.6)	450 (53.1)	184 (21.7)	41 (4.9)	337 (39.8)	510 (60.2)
Chronic bronchitis	17 (5.8)	21 (7.2)	195 (66.3)	53 (18.0)	8 (2.7)	105 (35.7)	189 (64.3)
Cold/ Chest cold	408 (24.2)	219 (13.0)	693 (41.1)	296 (17.5)	71 (4.2)	970 (57.5)	717 (42.5)
Flu/ Pneumonia/ Ear infection	126 (29.9)	49 (11.6)	154 (36.5)	74 (17.5)	19 (4.5)	272 (64.5)	150 (35.5)
Emphysema	8 (3.1)	16 (6.2)	194 (75.2)	29 (11.2)	11 (4.3)	88 (34.1)	170 (65.9)
Stomach/ Intestinal illness	176 (21.7)	89 (11.0)	388 (47.8)	121 (14.9)	37 (4.6)	391 (48.2)	420 (51.8)
Diabetes	247 (19.5)	148 (11.7)	503 (39.7)	314 (24.7)	56 (4.4)	595 (46.9)	673 (53.1)
High cholesterol	478 (14.6)	339 (10.3)	1763 (53.9)	567 (17.3)	127 (3.9)	1450 (44.3)	1824 (55.7)
Thyroid problem	96 (12.9)	66 (8.8)	477 (63.9)	85 (11.4)	22 (3.0)	300 (40.2)	446 (59.8)
Gout	31 (6.4)	21 (4.3)	292 (60.6)	115 (23.9)	23 (4.8)	184 (38.2)	298 (61.8)
Obesity	732 (18.7)	424 (10.8)	1864 (47.6)	680 (17.3)	219 (5.6)	1825 (46.6)	2094 (53.4)
Kidney stone	120 (12.7)	105 (11.1)	590 (62.5)	103 (10.9)	26 (2.8)	388 (41.1)	556 (58.9)
Failing kidney	45 (16.3)	38 (13.7)	124 (44.8)	58 (20.9)	12 (4.3)	122 (44.0)	155 (56.0)
Anemia	70 (14.9)	57 (12.1)	219 (49.5)	107 (22.7)	18 (3.8)	213 (45.2)	258 (54.8)
Liver condition	29 (19.6)	19 (12.8)	71 (48.0)	18 (12.2)	11 (7.4)	71 (48.0)	77 (52.0)
Heart attack	48 (10.3)	32 (6.9)	299 (64.0)	73 (15.6)	15 (3.2)	177 (37.9)	290 (62.1)
CHD	50 (11.5)	28 (6.4)	299 (68.7)	41 (9.4)	17 (4.0)	164 (37.7)	271 (62.3)
Stroke	33 (8.4)	26 (6.6)	228 (58.0)	91 (23.2)	15 (3.8)	156 (39.7)	237 (60.3)
Anxious	1056 (18.9)	643 (11.5)	2728 (48.7)	937 (16.7)	237 (4.2)	2572 (45.9)	3029 (54.1)
Sleeping trouble	305 (11.9)	237 (9.2)	1478 (57.6)	453 (17.7)	93 (3.6)	1089 (42.4)	1477 (57.6)
Depression	24 (19.0)	11 (8.7)	70 (55.6)	17 (13.5)	4 (3.2)	51 (40.5)	75 (59.5)
Cancer	72 (7.0)	49 (4.8)	750 (73.4)	128 (12.5)	23 (2.3)	345 (33.8)	677 (66.2)
Arthritis	354 (12.1)	257 (8.8)	1696 (58.1)	534 (18.3)	79 (2.7)	1217 (41.7)	1703 (58.3)
Prostate disease	13 (25.5)	9 (17.6)	19 (37.3)	8 (15.7)	2 (3.9)	32 (62.7)	19 (37.3)

### Characteristics of the study population and distributions of CRP and 25(OH)D

Distributions of CRP were significantly different (*P* < 0.05) in gender, examination period and race, and distributions of 25(OH)D were statistically significant (*P* < 0.05) in examination period and race. Characteristics of the study population and distributions of CRP and 25(OH)D concentrations were shown in Table 2.

**Table 2:** Distributions of CRP and 25(OH)D in gender, examination period and race.

Variables	CRP (mg/L)			25(OH)D (μg/L)		
	M [P_25_, P_75_]	Z	*P*	M [P_25_, P_75_]	Z	*P*
Gender		132.9	<0.001		0.01	0.94
Male	0.16[0.07, 0.38]			24[18, 30]		
Female	0.23[0.09, 0.54]			24 [17, 32]		
Examination period		15.7	<0.001		520.9	<0.001
Nov.1 ~ Apr.30	0.20[0.08, 0.49]			22[16, 28]		
May.1 ~ Oct.31	0.19[0.07, 0.43]			27[20, 33]		
Race		121.8	<0.001		2081.0	<0.001
Mexican American	0.22[0.09, 0.48]			21 [16, 26]		
Other Hispanic	0.20[0.08, 0.45]			23 [17, 29]		
Non-Hispanic White	0.18[0.07, 0.43]			29[23, 35]		
Non-Hispanic Black	0.25[0.09, 0.58]			16 [12, 23]		
Other Races	0.12[0.04, 0.30]			21 [16, 27]		

### The relationship of CRP and 25(OH)D in 24 specific diseases

**Figure 1:**
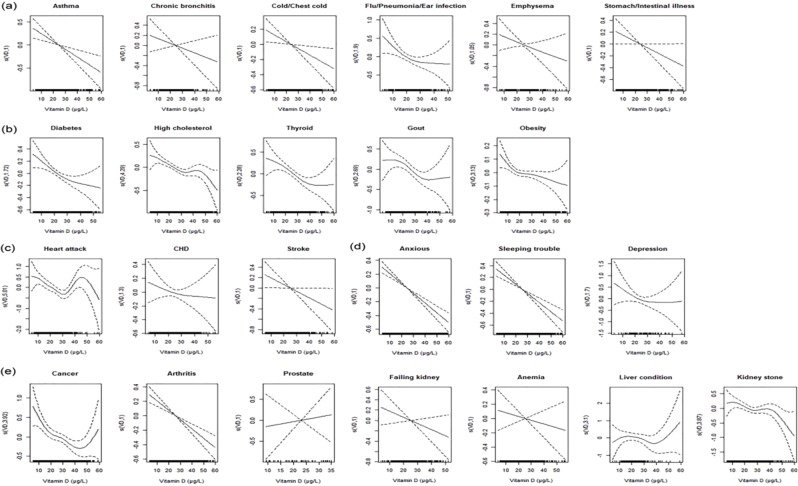
Associations between CRP and 25(OH)D in 24 specific diseases using GAM models, adjusted by age, gender, examination period and race. The vertical axis represents the smoothness function value, where the numbers in brackets represent EDF, and the dotted line represents 95% confidence interval ((**a**) respiratory and gastrointestinal diseases; (**b**) metabolic diseases; (**c**) cardiovascular and cerebrovascular diseases; (**d**) mental diseases; (**e**) other diseases).

As shown in Fig. 1, CRP was negatively associated with 25(OH)D generally, and the associations were different by diseases-specific. And the associated curves were similar in diseases of each category.

i) In general, in respiratory and gastrointestinal diseases (category “*a*”) and mental diseases (category “*d*”), CRP and 25(OH)D presented approximate linear associations, and the linear associations were stronger in mental diseases (category “*d*”);
ii) In metabolic diseases (category “*b*”), non-linear associations were presented in CRP and 25(OH)D. As 25(OH)D increasing, the downward tendency of the curves was slowing down. Taking diabetes for example, the decreasing tendency of the associated curve gradually got slow, especially when 25(OH)D was higher than 30 μg/L. Other diseases in metabolic diseases group (category “*b*”) also showed similar trends. Furthermore, the cut-off value of 25(OH)D in cancer was higher than in other diseases;
iii) In cardiovascular and cerebrovascular diseases (category “*c*”) and other diseases (category “*e*”), there was negative liner or non-liner associations between CRP and 25(OH)D generally.

### The parametric effects test of 25(OH)D in GAM

Table 3. showed the parametric effects test of 25(OH)D in GAM. Associations between CRP and 25(OH)D were statistically significant in majority diseases. Those minority diseases with nonsignificant associations may due to the small sample sizes of patients.

**Table 3:** The test for associations of CRP with 25(OH)D in GAM among 24 specific diseases.

Diseases	F	*P*	Diseases	F	*P*
**Asthma**	14.6	<0.001	Failing kidney	2.1	0.15
**Chronic bronchitis**	4.5	0.04	Anemia	0.8	0.38
**Cold/Chest cold**	10.8	0.001	Liver condition	0.2	0.68
Flu/Pneumonia/Ear infection	3.4	0.06	**Heart attack**	8.7	0.003
Emphysema	3.2	0.07	CHD	3.5	0.06
**Stomach/Intestinal illness**	4.8	0.03	**Stroke**	4.2	0.04
**Diabetes**	17.2	<0.001	**Anxious**	53.2	<0.001
**High cholesterol**	42.8	<0.001	**Sleeping trouble**	40.7	<0.001
**Thyroid problem**	11.5	<0.001	Depression	0.03	0.87
**Gout**	12.9	<0.001	**Cancer**	25.0	<0.001
**Obesity**	36.6	<0.001	**Arthritis**	50.2	<0.001
**Kidney stone**	7.0	0.008	Prostate disease	0.1	0.74

## Discussion

Vitamin D was considered to have potential beneficial effects on reducing inflammation and alleviating pain^7,8^. Thus, our study aimed to explore the associations of CRP with 25(OH)D in 24 specific diseases among adults of NHANES using GAM. Firstly, we found that 25(OH)D was negatively associated with CRP in general. And the associations were disease-specific and disease category-specific: in respiratory, gastrointestinal and mental diseases, the associations tended to be approximately linear. While in metabolic diseases, the associations were nonlinear, and the slope of the non-linear curve decreased with 25(OH)D, especially when 25(OH)D was less than 30 μg/ L. Secondly, the cut-off value of 25(OH)D in cancer was higher than that in other diseases.

25(OH)D was negatively associated with CRP in almost all diseases, which was similar to previous studies^15,18^. One of the possible reasons is vitamin D could inhibit the progression of inflammation by reducing the secretion of inflammatory cytokines such as IL-6, which could indirectly decrease the CRP level^19^. Another reason may be that CD8^+^ T cells could directly destroy the damaged or infected host cells, thereby reducing the amount of pro-inflammatory cells. Meanwhile, CD8^+^ T cells carried higher levels of vitamin D receptor (VDR) expression, whose function was regulated by vitamin D^20–22^, which may also indirectly down-regulate the CRP level.

In this study, associations of CRP with 25(OH)D differed in different disease categories. Similar negative associations, which presented approximate linear, were found in respiratory, gastrointestinal diseases, mental diseases and arthritis. Previous studies based on large populations showed that there was a strong dose-response association between lower vitamin D levels and increased risk of upper respiratory infection^23,24^. The potential mechanism for this may be that vitamin D, by activating VDR, had immunomodulatory effects on structural cells in airways^25^, while these cells could trigger the pathogenesis of asthma through complex interactions with inflammatory lymphocytes^26^. Besides, the immune regulation effect of vitamin D in asthma could both reduce airway hyper-responsiveness and inflammation, and contribute to the airway remodeling^27^.

Previous studied reported that vitamin D status was associated with the composition and function of the intestinal microbiome. In addition, vitamin D and VDR regulate the innate immune response to the microbiome^28,29^. Moreover, previous evidence suggested that vitamin D may be beneficial for autoimmune and allergic pathologies^30,31^.

Vitamin D may also have influence on emotion and depression^32,33^. This could attribute to inflammation, a well-known hypothesis mechanism for depression^34^. 25(OH)D was negatively associated with CRP in mental diseases. It is reported that low levels of vitamin D and VDR were significantly associated with higher levels of inflammatory markers, which could be attenuated by vitamin D supplementation^35^.

Further, we found nonlinearly negative associations of 25(OH)D with CRP in metabolic diseases. Previous studies also indicated that vitamin D might associate with the pathogenesis of type 2 diabetes mellitus (T2DM)^36^ and dyslipidemia^37^. It was reported that the supplementation of vitamin D may reduce systemic inflammation, that is, vitamin D supplementation had beneficial effect on the CRP levels^38,39^. The main potential mechanism of progression of T2DM was β cell dysfunction due to inflammatory stress and insulin resistance, while VDR was the key regulator of inflammation and beta cell survival^40^. There was evidence that the associations between 25(OH)D level and hypertriglyceridemia may be mediated through inflammation, as these associations disappeared after CRP as a covariate^41^. It was also suggested that obesity-related inflammation could lead to abnormal function of fat cells, elevate circulating levels of free fatty acid and ectopic lipid accumulation^42^.

In addition, in metabolic diseases, we found that the nonlinear associations of CRP with 25(OH)D had a weakening tendency as 25(OH)D increasing, which is similar with several previous studies. They found that the negative association between 25(OH)D and CRP would gradually decrease when 25(OH)D level was close to or higher than 21 μg /L^43,44^. This result indicated that, when the 25(OH)D level was higher than 30 μg/L, no additional clinical benefit was obtained, which was consistent with our study. It was also noteworthy that the cut-off value of 25(OH)D above-mentioned in cancer was higher than common diseases. Moreover, it was reported that higher 25(OH)D concentrations had benefits effect on the breast cancer, lung cancer and colorectal cancer^45–47^. However, the optimal doses of 25(OH)D concentration has not been determined yet^48,49^.

Thus, vitamin D supplementation might be always beneficial in respiratory, gastrointestinal and mental diseases, which may not so significant in metabolic diseases. Although metabolic diseases were not considered as inflammatory diseases traditionally, inflammatory components apparent promoting the disease process were increasingly taken into investigation^12^. The negative associations of CRP with vitamin D might provide greater potential therapeutic prospects for common diseases. Moreover, the supplementation of vitamin D in cancer should be more than that in common diseases for greater benefit.

Some limitations should be noted in present study. Firstly, the study was cross-sectional, we cannot draw a causal conclusion on the associations of CRP with 25(OH)D. Secondly, several unexplained variables might influence the associations of CRP with 25(OH)D cannot be taken into account. Thirdly, the complex sampling analysis was not involved in this study.

## Conclusion

In general, 25(OH)D was negatively associated with CRP, and the associations were disease-specific and disease category-specific. In respiratory, gastrointestinal and mental diseases, the associations tended to be approximately linear. While in metabolic diseases, the associations were nonlinear, and the slope of the nonlinear curve decreased with 25(OH)D, especially when 25(OH)D less than 30 μg/ L.

## Methods

### Sample

**Figure 2:**
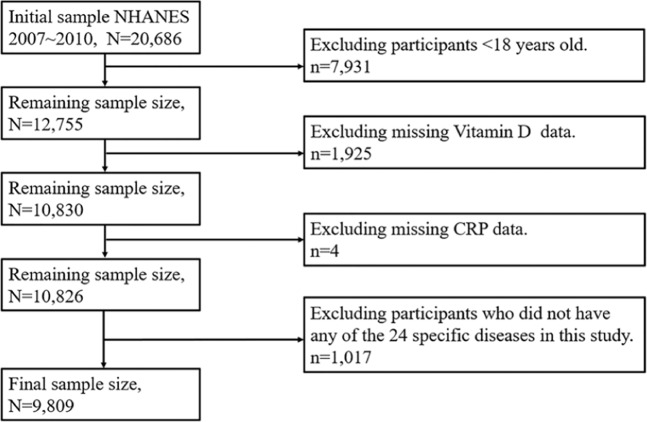
Flow chat for the study design and participants.

The NHANES, which were conducted by Centers for Disease Control and Prevention (CDC), aimed to assess the health status of the U.S. non-institutionalized civilian population. This survey utilized a complex probability sampling design, and collected information by standardized interviews, physical examinations and tests of biological samples^50,51^. A total of 20,686 subjects were enrolled in NHANES in 2007~2010. Our study focused on the relationship between CRP and 25(OH)D among adults in 24 specific diseases. For this purpose, the reluctant subjects who <18 years old (7,931 participants), unwilling to accept the measurement of 25(OH)D (1,925 participants) or CRP (4 participants) were excluded, and 1,017 participants who did not have any of 24 specific diseases were also excluded. As shown in Fig. 2, finally, a total of 9,809 participants who at least had one of diseases were involved in the study.

### Data measurement

NHANES database sociodemographic information included age, gender, examination period and race. Examination period indicated the time that the participant measured the 25(OH)D, which was classified into either November 1st through April 30th or May 1st through October 31st. Because it was rather cold in the northern states in winter, the data were collected in the northern states in summer and southern states in winter^52^. Race was coded in 5 categories: Non-Hispanic Whites, Non-Hispanic Blacks, Mexican Americans, other Hispanics, and other non-Hispanic race including non-Hispanic multiracial.

During the NHANES physical examination, weight and height were measured in a standardized fashion. Height and weight values were automatically transmitted from stadiometers and scales to the Integrated Survey Information System database. This database was designed to reduce data errors and contained age- and sex-specific edit ranges for each body-size measure on the basis of previous NHANES data. If an entry was outside this range, the recorder was alerted that the value was unusual and required to verify the measurement^53^. Body mass index (BMI) was calculated by the weight divided by height squared (kg/m^2^). Blood specimens were processed, stored and shipped to University of Washington, Seattle, WA. CRP was quantified by latex-enhanced nephelometry using a Behring Nephelometer^54^. The CDC used a standardized liquid chromatography-tandem mass spectrometry (LC-MS/MS) method traceable to measure 25(OH)D_3_, 25(OH)D_2_ and C3 epimer of 25(OH)D_3_. Vitamin D (variable LBXVIDMS, for NHANES 2007~2010) was defined as the sum of 25(OH)D_3_ and 25(OH)D_2_ excluded the C3 epimer of 25(OH)D_3_^55^.

Patients with the following diseases were defined as having been told by a doctor or other health professional: emphysema, diabetes, high cholesterol, thyroid problem, gout, kidney stone, failing kidney, anemia, heart attack, coronary heart disease (CHD), stroke, sleeping trouble, cancer, arthritis, prostate disease. Patients with asthma, chronic bronchitis and liver condition were defined as still having the disease at the time of the investigation. Patients with following diseases were defined as having these diseases started during those 30 days: cold/ chest cold, flu/ pneumonia/ ear infection, stomach or intestinal illness, anxious^56^. Patients with depression were defined as those whose Patient Health Questionnaire (PHQ-9) score ≥10, based on the participants’ symptoms over the past two weeks^57,58^. Obesity was defined those whose BMI ≥ 30 kg/m^2^ ^59^. In addition, patients with cancer were defined that who had cancer or a malignancy of any kind.

In this study, 24 specific diseases were divided into 5 categories: a. respiratory and gastrointestinal diseases, including asthma, chronic bronchitis, cold/ chest cold, flu/ pneumonia/ ear infection, emphysema and stomach/intestinal illness; b. metabolic diseases, including diabetes, high cholesterol, thyroid problem, gout and obesity; c. cardiovascular and cerebrovascular diseases, including heart attack, CHD and stroke; d. mental diseases, including anxious, sleeping trouble and depression; e. other diseases, including cancer, arthritis, prostate disease, failing kidney, anemia, liver condition and kidney stone.

### Statistical analysis

The x ± S and the M [P_25_, P_75_] were used to describe the distribution of continuous variables, and proportion was used to describe the distribution of categorical variables. Wilcoxon rank sum test was used to compare the continuous variables, and Rao-Scott-χ^2^ test was used to compare the categorical variables. CRP was log-transformed to obtain normal distributions for analysis, and back-transformed for representation in the tables. Age, gender, examination period and race were adjusted to examine the associations between log-CRP and 25(OH)D. GAM was also used to explore the associations of CRP with 25(OH)D. It was a generalization of the generalized additive model (GLM), which had great potential and flexibility in addressing non-linear relationships^60^. Two variables were linear associated when the estimated degrees of freedom (EDF) in GAM was equal to one. All statistical analyses were performed by R version 3.4.3, and the package “gam”^61^, “mgcv”^62^ and “glmnet”^63^ were used. Statistical significance was set at *P* < 0.05.

### Ethics approval and consent to participate

All NHANES protocols were approved by the National Center for Health Statistics’ Research Ethics Review Board, all participants signed a consent form before their participations and all research was performed in accordance with relevant guidelines/regulations. The statement of informed consent is openly available in https://www.cdc.gov/nchs/nhanes/biospecimens/participants.htm.

### Informed consent

Health information collected in the NHANES is kept in strictest confidence. During the informed consent process, survey participants are assured that data collected will be used only for stated purposes and will not be disclosed or released to others without the consent of the individual or the establishment in accordance with section 308(d) of the Public Health Service Act (42 U.S.C. 242 m). Only samples from participants who have consented for future research are stored in the NHANES Biospecimen Repository and are available to researchers.

## Data Availability

The data that support the findings of this study are openly available in https://www.cdc.gov/nchs/nhanes/. Information from NHANES is made available through an extensive series of publications and articles in scientific and technical journals. For data users and researchers throughout the world, survey data are available on the internet and on easy-to-use CD-ROMs.
